# Intraosseous Mucoepidermoid Carcinoma in the Mandible

**DOI:** 10.1155/2018/9348540

**Published:** 2018-12-17

**Authors:** George Borja de Freitas, Arthur José Barbosa de França, Stefanny Torres dos Santos, Miqueias Oliveira de Lima Júnior, Adilis Stepple da Fonte Neto, Paula Bernardon

**Affiliations:** ^1^Brazilian Association of Dentistry (ABO/PE), Street Dois Irmãos, 165, Apipucos, 13045755 Recife, PE, Brazil; ^2^Hospital Getúlio Vargas-Recife/PE—Brazilian Association of Dentistry (ABO/PE), Street Dois Irmãos, 165, Apipucos, 13045755 Recife, PE, Brazil; ^3^Hospital of Cancer of Pernambuco-Recife/PE—Brazilian Association of Dentistry (ABO/PE), Street Dois Irmãos, 165, Apipucos, 13045755 Recife, PE, Brazil; ^4^State University of Western Parana, Cascavel, PR, Brazil; ^5^Paranaense University, Cascavel, PR, Brazil; ^6^Dental Clinics-St. Ovalo Bilac, 1251 Cascavel, Paraná, Brazil

## Abstract

Although it is a rare neoplasm, intraosseous mucoepidermoid carcinoma is the most common and well-recognized intraosseous salivary gland tumor. Usually, it presents as an asymptomatic volume increase and most patients perceive the presence of the lesion within a year or less of evolution. They are more common in middle-aged adults and have a slight female predilection. They are three times more common in the mandible than in the maxilla and are most often found in the area of the molars and mandibular ramus. The most frequently present symptom is cortical bulging, although some lesions may be discovered as an accidental finding on radiographs. The main modality of treatment for patients with this neoplasm is radical surgical resection, offering a greater chance of cure than the more conservative procedures, such as enucleation or curettage, due to the great possibility of recurrence and tumor metastasis. This paper reports a rare case of intraosseous mucoepidermoid carcinoma occasionally discovered after panoramic radiography of the jaws, which was treated with segmental resection through hemimandibulectomy.

## 1. Introduction

Salivary carcinoma accounts for 3 to 4% of all head and neck cancers, and of these, mucoepidermoid carcinoma (MEC) is the most common type. MEC demonstrates highly variable clinical behavior, ranging from slow to indolent to locally aggressive and highly metastatic tumors. MEC occurs predominantly in the larger and parotid salivary glands. When it affects the minor salivary glands, it is most frequently found on the palate, followed by the retromolar gap, buccal mucosa, tongue, lips and floor of the mouth, sinuses, and larynx [1].

Intraosseous mucoepidermoid carcinoma is a rare neoplasm of the gnathic bones. Although theories have been proposed based on the neoplastic transformation of the epithelial mucosa of odontogenic cysts or ectopic salivary tissue, their origin is uncertain [2].

The main modality of treatment for patients with this neoplasm is radical surgical resection, offering a greater chance of cure than the more conservative procedures, such as enucleation or curettage. The rate of local recurrence associated with conservative treatment is 40%, contrasting with a rate of 13% for the more radical treatment. Metastases have been reported in about 12% of the cases [3].

The aim of the present article is to report a case of a patient with low-grade mandibular intraosseous mucoepidermoid carcinoma who was diagnosed after routine consultation and subsequently treated with resection through hemimandibulectomy.

## 2. Case Presentation

**Figure 1:**
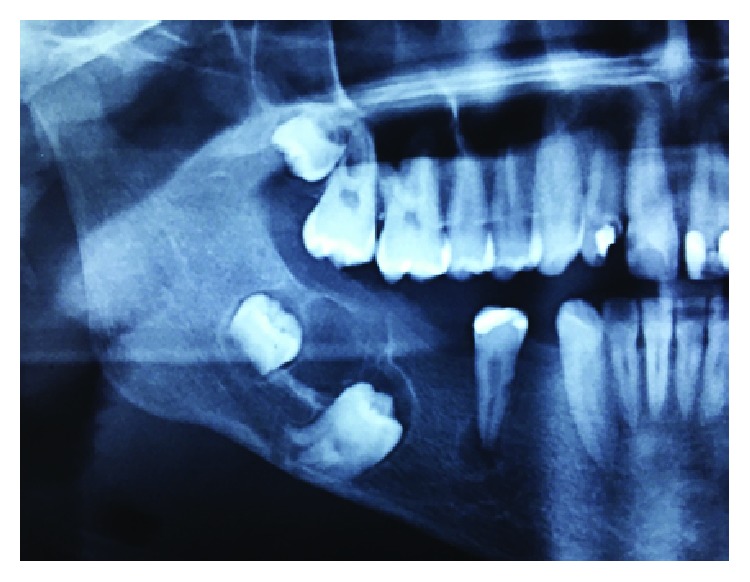
Initial tomographic appearance of the lesion.

**Figure 2:**
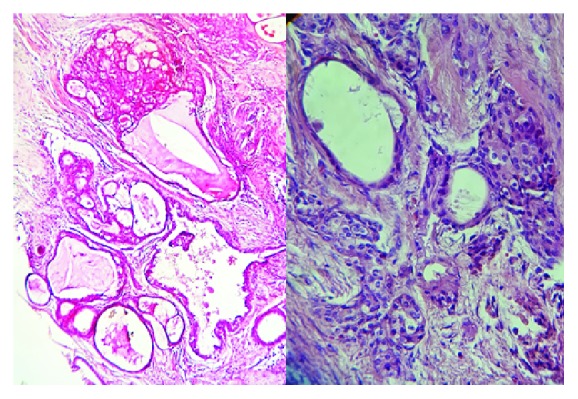
Histological appearance evidencing prominent cystic formation, minimal cellular atypia, and relatively high proportion of mucous cells.

A 16-year-old male patient was referred by the orthodontist after a radiolucent lesion on the mandible was discovered after a panoramic X-ray of the jaws during orthodontic treatment, with approximately 4 months of evolution (Figure 1). At the extraoral physical examination, there were no signs of increased volume and/or facial asymmetry; at the intraoral examination, the lesion presented with lingual cortical expansion, mucosa with normal coloration, no dental displacement, and absence of painful symptomatology with negative aspiration puncture. At tomographic examination, the patient presented a multilocular hypodense image in the body region and right mandibular angle, associated with retained teeth 47 and 48, which was initially suggested to be an odontogenic cyst or another tumor. The patient did not present comorbidities and/or basic, nonsmoking, and nonalcoholic diseases. An incisional biopsy was performed on the affected area, and a histopathological report of low-grade intraosseous mucoepidermoid carcinoma was prepared (Figure 2). Immunohistochemical analysis of the lesion was performed through the CK-7 marker to confirm the diagnosis. According to the pathologist, mucin staining was performed for histology.

**Figure 3:**
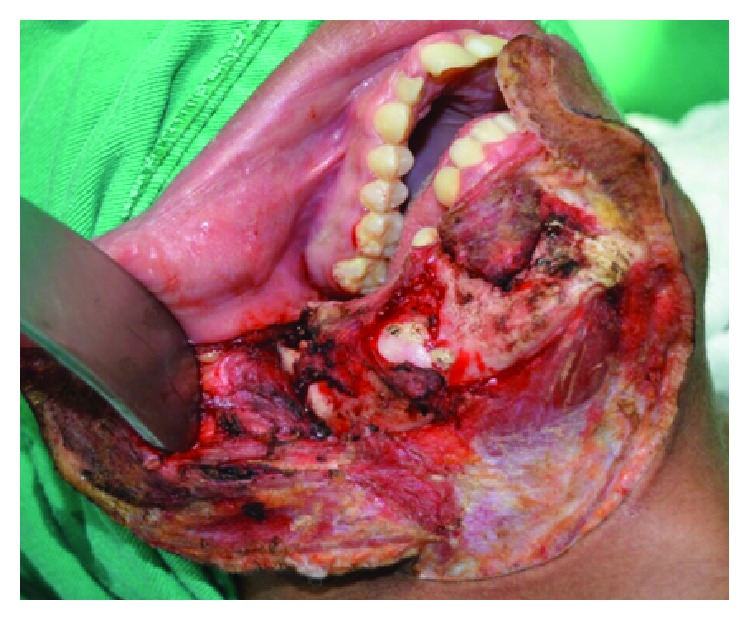
Surgical access for hemimandibulectomy.

**Figure 4:**
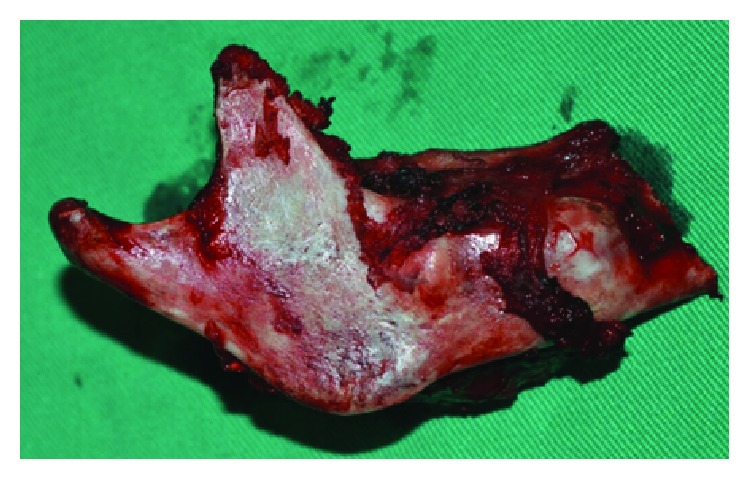
Surgical specimen removed after hemimandibulectomy.

**Figure 5:**
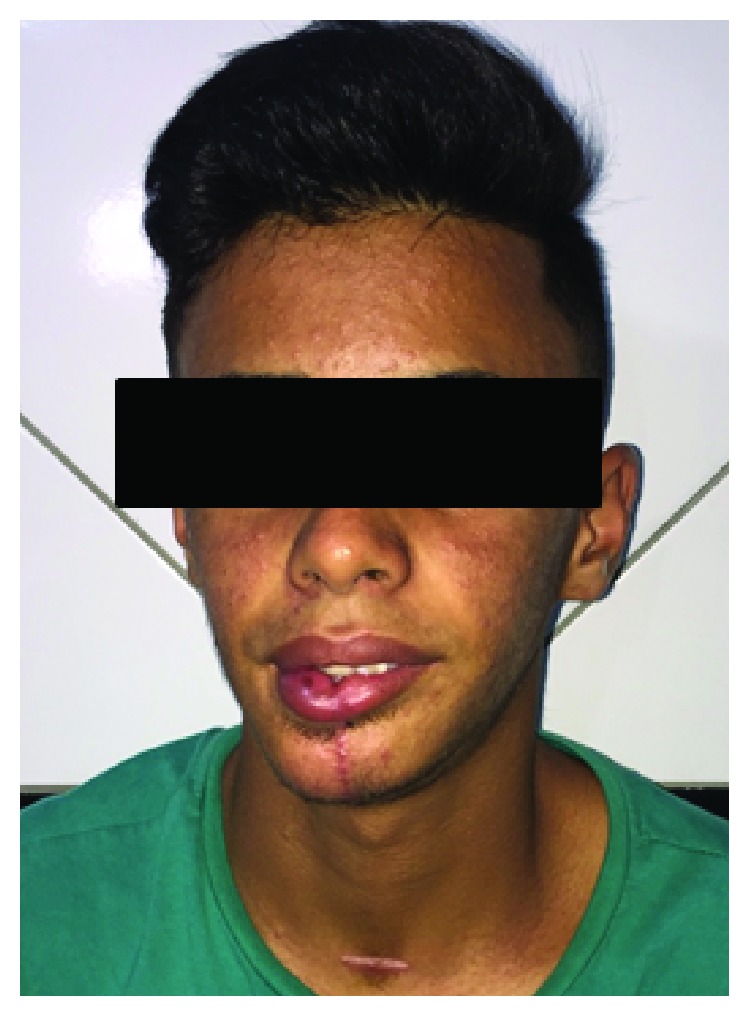
Clinical appearance after 2 years of preservation.

The surgical planning was segmental resection through the right hemimandibulectomy with a safety margin, from the right submandibular access with extension to the lower lip (Figures 3 and 4). Subsequently, the patient was submitted to radiotherapy in order to mitigate the chances of the lesion. The patient was followed for 2 years and has had no clinical evidence of relapse and/or metastasis (Figure 5).

## 3. Discussion

Mucoepidermoid carcinoma (MEC) usually arises from larger or smaller salivary glands and makes up 5% to 10% of all salivary gland tumors, whereas intraosseous glands comprise only 2% to 3% of all MEC and occur more frequently in the posterior region of the mandible [4].

Although the exact pathogenesis of this lesion is unknown, there are several current theories of its origin. The following may represent origins for these lesions: (a) ectopic salivary gland tissue: remnants of embryonic salivary glands trapped within the bone; (b) transformation of mucous cells found in odontogenic cysts; and (c) maxillary sinuses or submucosal and mucosal glands with intraosseous extension [5]. More recently, intraosseous salivary tissue has been found to be present in 0.3% of the bone specimens of all maxillary bones studied by Bouquot et al. [2], providing new evidence for the origin of intraosseous salivary carcinomas. Although its etiology is questionable, mandibular intraosseous MEC is an accepted entity [6].

Traditionally, mucoepidermoid carcinomas have been classified into three histopathological grades using the following criteria: quantity of cystic formation, degree of cellular atypia, and relative number of mucous, epidermoid, and intermediate cells. Low-grade tumors exhibit prominent cystic formation, minimal cellular atypia, and a relatively high proportion of mucosal cells. High-grade tumors consist of solid islands of squamous and intermediate cells, which may demonstrate considerable pleomorphism and mitotic activity. Mucus-producing cells may be infrequent, and sometimes, it may be difficult to distinguish the tumor from squamous cell carcinoma. However, those of intermediate-grade exhibit characteristics that are located between low- and high-grade tumors [3].

Intraosseous mucoepidermoid carcinomas are more common in middle-aged adults and have a slight preference for females. They are three times more common in the mandible than in the maxilla and are most often found in the area of the molars and mandibular ramus. The most frequently present symptom is cortical bulging, although some lesions may be discovered as an accidental finding on radiographs. Pain, trismus, and paresthesia are symptoms reported less frequently in these lesions. Metastases have been reported in 12% of the cases, often as a result of local tumor recurrence, mainly for regional lymph nodes and occasionally for the ipsilateral clavicle, lung, and brain. About 10% of the patients evolve to death [3, 7].

Imaging plays an important role in the detection and differentiation of MEC because of its sclerotic periphery and mixed internal structure, consisting of a unilocular and/or multilocular pattern with imaging characteristics similar to those of other lesions, including ameloblastoma, glandular odontogenic cyst, and keratocystic odontogenic tumour (Table 1). Panoramic radiography and conventional computed tomography (CT) are routinely used as diagnostic tools for evaluating the maxillofacial area [8].

It is described as a radiolucent image with well-defined scleral periphery and numerous small loculations. The presence of tooth dislocation and root resorption are common findings. Its aggressive behavior is revealed by cortical bone perforation and extension to surrounding soft tissues [9]. When the correlation between the clinical and histopathological diagnosis was analyzed, only 12.5% of the cases presented a correlation, so the final diagnosis should be based on clinical, radiographic, and histopathological characteristics [10]. Studies suggest that fine needle aspiration (FNA) is considered to be effective for high-grade or intermediate-grade but unsatisfactory for low-grade EMBs [4].

Surgery is the main form of treatment. In a review of 64 patients, Brookstone and Huvos observed 40% relapses after conservative surgical modalities such as enucleation, curettage, marsupialization, and marginal resection with or without adjuvant therapy, whereas in the group treated by radical methods such as segmental resection with or without adjuvant treatment associated with the neck, only 4% relapsed. Adjuvant therapy, such as radiotherapy and/or chemotherapy, is recommended for high-grade tumors [11, 12].

Lee et al. [13] provided both experimental and preclinical evidence that specificity protein 1 is an important regulator of MEC growth and is an effective target of apoptotic therapy. Dibenzylideneacetone significantly inhibited specificity protein 1 through the regulation of protein stability and modulated the expression of the proapoptotic proteins, Bim and truncated Bid, which are dependent on Sp1 protein [13].

## 4. Conclusions

Although it is a rare neoplasm, intraosseous mucoepidermoid carcinoma is the most common and well-recognized intraosseous salivary gland tumor. Metastases have been reported in 12% of the cases, and about 10% of the patients evolve to death, often as a result of local tumor recurrence. The present case shows that the clinical significance of these tumors should never be underestimated, emphasizing the importance of radical treatment, adjuvant therapy, and a careful histopathological evaluation of all excised tissue, so that such neoplastic transformation can be effectively identified and treated.

## Figures and Tables

**Table 1:** Main pathologies for differential diagnosis.

Lesion	Type	Gender	Mean age (years)	Anatomic location	Clinical signs	Radiological appearance
Central mucoepidermoid carcinoma	Malignant	Female	40	Mandible	Slow growth and painless mass with cortical expansion	Radiolucent (uni-/multilocular) with well-defined borders Tooth resorption
Cystic adenoid carcinoma	Malignant	Female	40	Palate	Slow growth and painful mass	Bone destruction
Glandular odontogenic cyst	Benign	No predilection	49	Mandible	Small lesions (asymptomatic) Large lesions (bone expansion, pain, and paresthesia)	Radiolucent (uni-/multilocular) with well-defined margins and sclerotic borders
Squamous cell carcinoma	Malignant	Male	40	Mandible	Lesion with a centrally depressed and irregularly ulcerated region	Radiolucent area with poorly defined borders (moth-eaten aspect)
Ameloblastoma	Benign	No predilection	30-70	Mandible	Asymptomatic, but may show bone expansion	Radiolucent (uni-/multiocular) Cortical expansion Tooth resorption
Keratocyst	Benign	Male	10-40	Mandible	Usually asymptomatic and with no cortical expansion	Radiolucent area with well-defined margins. Anterior-posterior growth through the medullary space

## References

[1] Coca-Pelaz A., Rodrigo J. P., Triantafyllou A. (2015). Salivary mucoepidermoid carcinoma revisited. *European Archives of Oto-Rhino-Laryngology*.

[2] Bouquot J. E., Gnepp D. R., Dardick I., Hietanen J. H. P. (2000). Intraosseous salivary tissue: jawbone examples of choristomas, hamartomas, embryonic rests, and inflammatory entrapment: another histogenetic source for intraosseous adenocarcinoma. *Oral Surgery, Oral Medicine, Oral Pathology, Oral Radiology, and Endodontology*.

[3] Neville B. W., Damm D. D., Allen C. M., Bouquot J. E. (2009). *Patologia Oral & Maxilofacial*.

[4] Bell D., Lewis C., El-Naggar A. K., Weber R. S. (2016). Primary intraosseous mucoepidermoid carcinoma of the jaw: reappraisal of the MD Anderson Cancer Center experience. *Head & Neck*.

[5] Johnson B., Velez I. (2008). Central mucoepidermoid carcinoma with an atypical radiographic appearance. *Oral Surgery, Oral Medicine, Oral Pathology, Oral Radiology, and Endodontology*.

[6] Pires F. R., Almeida O. P., Lopes M. A., Perez D. E. C., Kowalski L. P. (2003). Central mucoepidermoid carcinoma of the mandible: report of four cases with long-term follow-up. *International Journal of Oral and Maxillofacial Surgery*.

[7] Simon D., Somanathan T., Ramdas K., Pandey M. (2003). Central mucoepidermoid carcinoma of mandible—a case report and review of the literature. *World Journal of Surgical oncology*.

[8] Costa A. L. F., Ferreira T. L. D., Soares H. A., Nahas-Scocate A. C. R., Montesinos G. A. P., Braz-Silva P. H. (2017). Cone beam computed tomography diagnostic imaging of intra-osseous mucoepidermoid carcinoma in the mandible. *Journal of Clinical and Experimental Dentistry*.

[9] Chan K., Pharoah M., Lee L., Weinreb I., Perez-Ordonez B. (2013). Intraosseous mucoepidermoid carcinoma: a review of the diagnostic imaging features of four jaw cases. *Dento Maxillo Facial Radiology*.

[10] He Y., Wang J., Fu H. H., Zhang Z. Y., Zhuang Q. W. (2012). Intraosseous mucoepidermoid carcinoma of jaws: report of 24 cases. *Oral Surgery, Oral Medicine, Oral Pathology, Oral Radiology*.

[11] Nance M. A., Seethala R. R., Wang Y. (2008). Treatment and survival outcomes based on histologic grading in patients with head and neck mucoepidermoid carcinoma. *Cancer*.

[12] Brookstone M. S., Huvos A. G. (1992). Central salivary gland tumors of the maxilla and mandible: a clinicopathologic study of 11 cases with an analysis of the literature. *Journal of Oral and Maxillofacial Surgery*.

[13] Lee H. E., Choi E. S., Jung J. Y., You M. J., Kim L. H., Cho S. D. (2014). Inhibition of specificity protein 1 by dibenzylideneacetone, a curcumin analogue, induces apoptosis in mucoepidermoid carcinomas and tumor xenografts through Bim and truncated Bid. *Oral Oncology*.

